# Supplementary material for: Modeling glioblastoma heterogeneity as a dynamic network of cell states
Source: Mol Syst Biol. 2021 Sep 16;17(9):e10105. doi: 10.15252/msb.202010105 (PMC8444284; doi:10.15252/msb.202010105)
Supplement: Supplementary file 6 — Source Data for Figure 5 [file MSB-17-e10105-s004.zip › Figure5A_sourcedata/GSEA_3017/nosigdb_stateC.GseaPreranked.1622795002785/RICHARDS_NEFTEL_CELL_2019_G1_S.html]

Details for gene set RICHARDS\_NEFTEL\_CELL\_2019\_G1\_S[GSEA]

|  || Dataset | state13017 |
| Phenotype | NoPhenotypeAvailable |
| Upregulated in class | na\_pos |
| GeneSet | RICHARDS\_NEFTEL\_CELL\_2019\_G1\_S |
| Enrichment Score (ES) | 0.7373106 |
| Normalized Enrichment Score (NES) | 3.174816 |
| Nominal p-value | 0.0 |
| FDR q-value | 0.0 |
| FWER p-Value | 0.0 |
Table: GSEA Results Summary

  

Fig 1: Enrichment plot: RICHARDS\_NEFTEL\_CELL\_2019\_G1\_S      
 Profile of the Running ES Score & Positions of GeneSet Members on the Rank Ordered List

  

| PROBE | GENE SYMBOL | GENE\_TITLE | RANK IN GENE LIST | RANK METRIC SCORE | RUNNING ES | CORE ENRICHMENT || 1 | HIST1H4C |  |  | 9 | 0.603 | 0.0792 | Yes |
| 2 | CCNE2 |  |  | 15 | 0.570 | 0.1600 | Yes |
| 3 | RRM2 |  |  | 19 | 0.542 | 0.2400 | Yes |
| 4 | GMNN |  |  | 38 | 0.453 | 0.2802 | Yes |
| 5 | RAD51AP1 |  |  | 46 | 0.435 | 0.3365 | Yes |
| 6 | PCNA |  |  | 48 | 0.435 | 0.4031 | Yes |
| 7 | RRM1 |  |  | 62 | 0.386 | 0.4413 | Yes |
| 8 | CDCA5 |  |  | 81 | 0.361 | 0.4670 | Yes |
| 9 | GINS2 |  |  | 83 | 0.360 | 0.5218 | Yes |
| 10 | DUT |  |  | 87 | 0.356 | 0.5725 | Yes |
| 11 | MCM7 |  |  | 91 | 0.352 | 0.6227 | Yes |
| 12 | FEN1 |  |  | 98 | 0.342 | 0.6661 | Yes |
| 13 | MCM3 |  |  | 114 | 0.324 | 0.6912 | Yes |
| 14 | CENPK |  |  | 139 | 0.299 | 0.6968 | Yes |
| 15 | ASF1B |  |  | 143 | 0.291 | 0.7373 | Yes |
| 16 | MCM4 |  |  | 189 | 0.259 | 0.7005 | No |
Table: GSEA details [plain text format]

  

Fig 2: RICHARDS\_NEFTEL\_CELL\_2019\_G1\_S: Random ES distribution      
 Gene set null distribution of ES for **RICHARDS\_NEFTEL\_CELL\_2019\_G1\_S**
